# The p75 neurotrophin receptor regulates cranial irradiation-induced hippocampus-dependent cognitive dysfunction

**DOI:** 10.18632/oncotarget.16492

**Published:** 2017-03-23

**Authors:** Xin Ding, Hao-Hao Wu, Sheng-Jun Ji, Shang Cai, Pei-Wen Dai, Mei-Ling Xu, Jun-Jun Zhang, Qi-Xian Zhang, Ye Tian, Quan-Hong Ma

**Affiliations:** ^1^ Department of Radiotherapy & Oncology, The Second Affiliated Hospital of Soochow University, Suzhou, China; ^2^ Institute of Radiotherapy & Oncology, Soochow University, Suzhou, China; ^3^ Suzhou Key Laboratory for Radiation Oncology, Suzhou, China; ^4^ Department of Radiotherapy and Oncology, Nanjing Medical University Affiliated Suzhou Hospital, Suzhou, China; ^5^ Jiangsu Key Laboratory of Translational Research and Therapy for Neuro-Psycho-Diseases and Institute of Neuroscience, Soochow University, Suzhou, China

**Keywords:** hippocampus, cognitive dysfunction, neurogenesis, dendritic spine, p75^NTR^

## Abstract

Cognitive deficits, characterized by progressive problems with hippocampus-dependent learning, memory and spatial processing, are the most serious complication of cranial irradiation. However, the underlying mechanisms remain obscure. The p75 neurotrophin receptor (p75^NTR^) is involved in a diverse arrays of cellular responses, including neurite outgrowth, neurogenesis, and negative regulation of spine density, which are associated with various neurological disorders. In this study, male Sprague-Dawley (SD) rats received 10 Gy cranial irradiation. Then, we evaluated the expression of p75^NTR^ in the hippocampus after cranial irradiation and explored its potential role in radiation-induced synaptic dysfunction and memory deficits. We found that the expression of p75^NTR^ was significantly increased in the irradiated rat hippocampus. Knockdown of p75^NTR^ by intrahippocampal infusion of AAV8-shp75 ameliorated dendritic spine abnormalities, and restored synapse-related protein levels, thus preventing memory deficits, likely through normalization the phosphor-AKT activity. Moreover, viral-mediated overexpression of p75^NTR^ in the normal hippocampus reproduced learning and memory deficits. Overall, this study demonstrates that p75^NTR^ is an important mediator of irradiation-induced cognitive deficits by regulating dendritic development and synapse structure.

## INTRODUCTION

Radiotherapy, a commonly applied treatment for brain tumors, may cause serious brain injury, especially damages to the hippocampus, an area important for cognition. Radiation causes both anatomical and functional changes, which result in impaired hippocampus-dependent learning, memory and spatial processing abilities [1–3]. Clinical studies have reported that radiation-induced cognitive dysfunction occurs in up to 50% of long-term brain tumor survivors [4]. Although altered hippocampal neurogenesis, abnormal neurotrophin levels and aberrant neuroinflammation have been proposed as underlying mechanisms [5], little is known about the precise molecular pathways involved in radiation-induced learning and memory disturbances.

Neurotrophins and their receptors play pivotal roles in brain development and in maintaining the physiological functions of the nervous system [6]. Brain-derived neurotrophic factor (BDNF) is the most abundant neurotrophin in the brain, and it has received increasing attention [7–9]. BDNF has two receptors, TrkB and p75^NTR^. BDNF modulates cognitive outcome by regulating hippocampal neurogenesis and synaptic plasticity via its interactions with TrkB receptors [10–12]. However, the interactions between BDNF and p75^NTR^ are poorly understood.

P75^NTR^, a member of the tumor necrosis factor receptor superfamily, is widely expressed in the developing central nervous system (CNS) [13], and is involved in neuronal survival, neurite outgrowth [14, 15] and synaptic plasticity [16]. P75^NTR^ may be associated with several neurological disorders. The expression of p75^NTR^ is downregulated in the adult CNS and re-expressed after injury [17, 18]. Increased levels of p75^NTR^ have been detected in the cortex and hippocampus of Alzheimer's disease (AD) patients [19], which is characterized by cognition decline. Consistently, treatment with small-molecule ligands of p75^NTR^ prevent cognitive decline in AD mouse models [20, 21]. In addition, p75^NTR^ mediates synaptic plasticity and thus cognitive dysfunction in Huntington's disease [22]. These lines of evidence suggest essential roles for p75^NTR^ in mediating cognition in neurological disorders, possibly including radiation-induced cognitive dysfunction. In the present study, we observed increased levels of p75^NTR^ in the hippocampus of irradiated rats, which exhibits deficit cognition and decreased synaptic plasticity. We further found that overexpression of p75^NTR^ in the hippocampus of rats without irradiation results in similar abnormalities to those in irradiated rats. Moreover, the deficits in cognition and synaptic plasticity of irradiated rats were rescued by knock down of p75^NTR^ in the hippocampus. We conclude that p75^NTR^ plays potential roles in irradiation-induced cognitive dysfunction by mediating synaptic plasticity.

## RESULTS

### The expression and location of p75^NTR^ in the irradiated rat hippocampus

**Figure 1:**
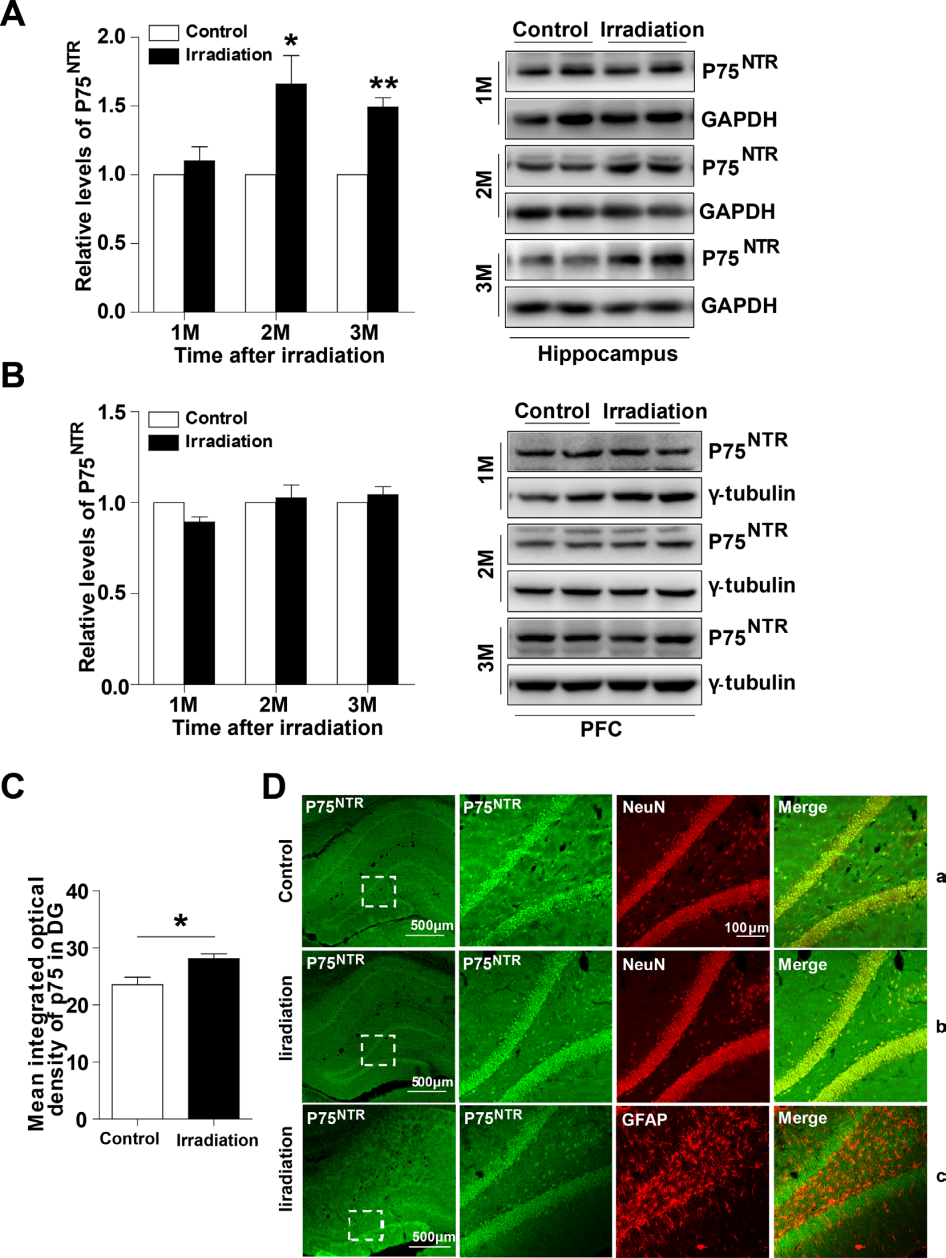
The expression and location of p75^NTR^ in the irradiated rat hippocampus Western blot analysis of p75^NTR^ in the hippocampus **(A)** and PFC **(B)** at 1, 2 and 3 months post-irradiation. Right: Representative immunoblots. Quantitative analysis reveals an increase mean integrated optical density of p75^NTR^ in the DG region at 2 months post-irradiation **(C)**. Representative confocal microscopy images (low magnification) showing the expression pattern of p75^NTR^ in rat hippocampus **(D)**. Magnified images (right) show colocalization between p75^NTR^ and NeuN in normal rat hippocampus (D-a) and irradiated rat hippocampus (D-b), p75^NTR^ and GFAP (D-c) in the DG region. Data are presented as mean ±SEM. *p<0.05; **p<0.01; ***p<0.001. n=5/group.

To analyze the potential role of p75^NTR^ in radiation-induced cognitive dysfunction, we first examined expression of p75^NTR^ levels in the post-irradiation rats. Western blot analysis revealed increased levels of p75^NTR^ in the hippocampus of irradiated rats at 2 months and 3 months, but not 1 month after irradiation compared with controls (Figure 1A). In contrast, the levels of p75^NTR^ in the dorsolateral prefrontal cortex (PFC) remained unchanged upon irradiation (Figure 1B). These results suggest a pathological function of p75^NTR^ in radiation-induced cognitive dysfunction. To address p75^NTR^ localization, we used an immunofluorescence staining technique. Confocal analysis of brain sections showed that p75^NTR^ immunoreactivity as assessed by punctate staining, was more dispersed in the granular cell layer of the dentate gyrus (DG) as well as in the stratum oriens within the CA1 and CA3 regions of the hippocampus. According with our biochemical data, p75^NTR^ immunoreactivity in irradiated rats was higher than in controls (Figure 1C). When subcellular localization was analyzed in the DG region, we found that p75^NTR^ immunoreactivity colocalized with NeuN, a specific marker for neurons, suggesting that p75^NTR^ was expressed by hippocampal neurons before and after irradiation (Figure 1D-a and 1D-b). Since p75^NTR^ is also expressed by astrocytes, especially after neuronal damage, coimmunostaining with the astrocytic marker GFAP was performed in hippocampal slices from irradiated animals. Interestingly, lack of colocalization between p75^NTR^ and GFAP was found, indicating that astrocytes in irradiated rat hippocampus did not overexpress p75^NTR^ (Figure 1D-c). These findings suggest that neuronal p75^NTR^ upregulation underlies radiation-induced hippocampal dysfunction.

### Intrahippocampal infusion of AAV-GFP-p75^NTR^ in normal rats reproduces memory impairments

**Figure 2:**
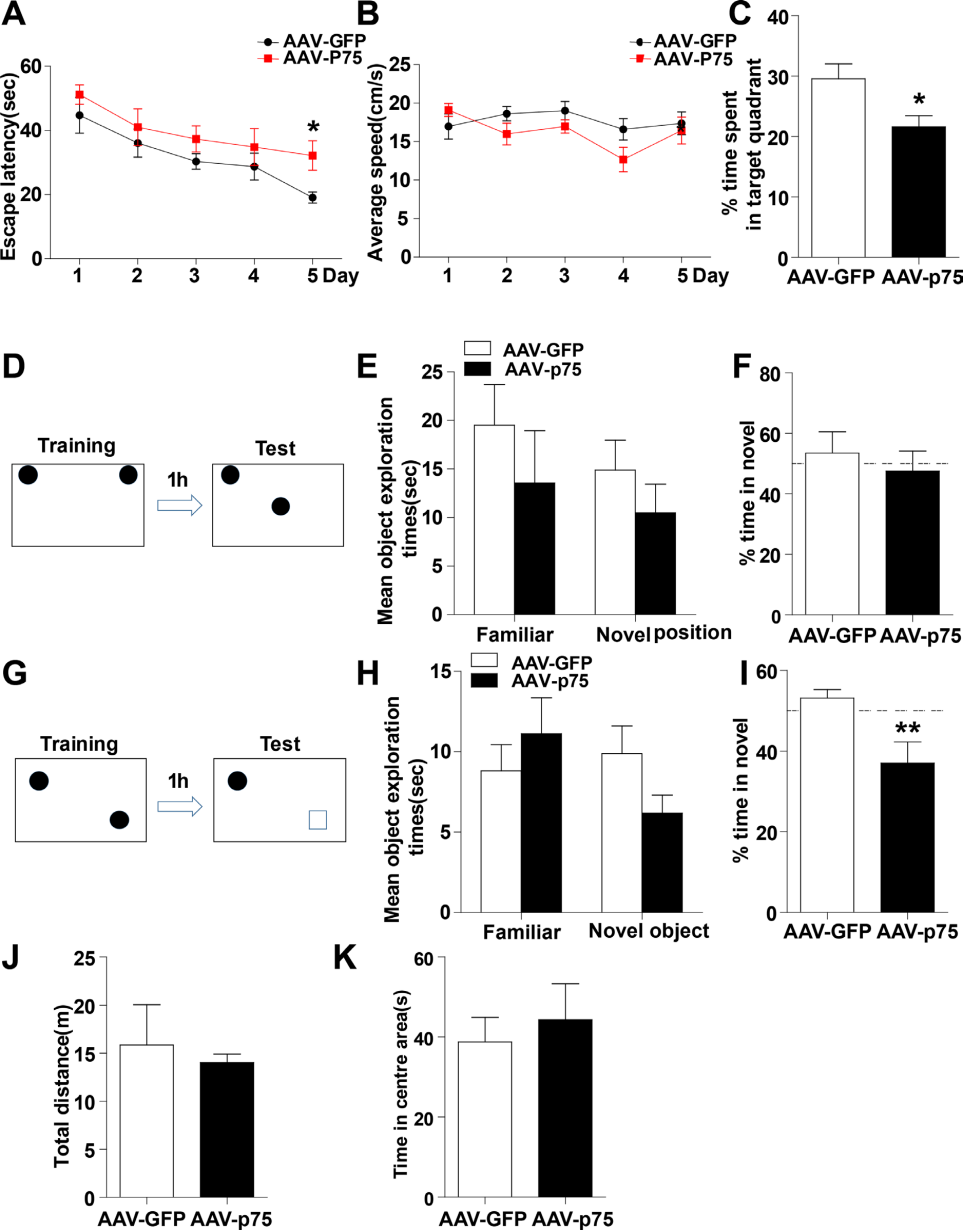
Intrahippocampal infusion of AAV-p75 in normal rats reproduce the cognitive deficits **(A-C)** *Morris water maze test*. Comparison of the escape latencies (A), mean swimming speed (B) and the percentage of target quadrant exploring time in probe test (C) between the AAV-GFP and AAV-P75 groups. **(D-F)** *Object location recognition test*. Diagram of the object location recognition task (D); The graph shows the object exploration during the test phase (E); Both AAV-GFP and AAV-p75 rats spent more time exploring a novel object location (F). **(G-I)** *Novel object recognition test*. Diagram of the object recognition task (G); The graph shows the object exploration during the 5 min test phase (H); The AAV-p75 rats did not display any preference for an object placed to a novel object (I). **(J-K)** *Open field test*. No significant differences were detected in total distance travelled test (J), and percent time travelled in the centre of the open files (K) between AAV-GFP and AAV-p75 groups. All data are presented as mean ±SEM. *p<0.05; **p<0.01. n= 10-15/group.

To further estimate whether the aberrant increase in hippocampal p75^NTR^ levels could contribute to memory impairments, we next tested whether overexpression of p75^NTR^ only in the normal rat hippocampus was able to mimic radiation-induced memory deficits. First, we analyzed whether the virus serotype AAV8 was able to infect glial and/or neuronal cells. Confocal microscopy analysis showed that slices from normal rats injected with AAV8-GFP-p75^NTR^, the virus efficiently transduces hippocampal neurons but not astrocytes, indicating that the 8 serotype is highly specific for neuronal cells within the hippocampus (Supplementary Figure 1A). Then, we confirmed that p75^NTR^ expression is greater in the hippocampus of AAV-p75 rats than in AAV-GFP animals by Western blot analysis (Supplementary Figure 1B). When memory function was evaluated, normal rats overexpressing p75^NTR^ showed spatial and nonspatial memory impairments. The Morris water maze is a test of spatial learning for rodents, and all rats improved their performance on days 1-4 during the place navigation test. While on days 5, the AAV-p75 group spent longer latency time than in AAV-GFP group (Figure 2A). The changes in escape latency were not due to the differences in swimming speed, which were no difference between the groups (Figure 2B). In the spatial probe test, the AAV-GFP group showed memory retention and spent significantly more time in the target quadrant than the AAV-p75 group (Figure 2C). Novel location and novel object recognition tests were used to evaluate hippocampus- and cortex-dependent spatial and nonspatial learning and memory. Both groups spent more time exploring the object in the novel location, with no significant difference between groups (Figure 2D-2F). After the novel location trial, rats were tested for novel object recognition. During the training sessions, both groups of rats spent the same amount of time exploring the two identical objects. However, the AAV-p75 group failed to distinguish the familiar object and a novel object (Figure 2G-2I). We also assessed anxiety levels and sensorimotor function of all rats by an open field test. Our behavioral analyses showed no differences in exploratory activity and measures of anxiety between groups (Figure 2J and 2K). These experiments indicate that overexpression of p75^NTR^ in the normal rat hippocampus reproduces memory deficits, providing evidence for the role of p75^NTR^ in radiation-induced cognitive dysfunction.

### Knockdown of p75^NTR^ expression in the irradiated rat hippocampus prevents memory impairments

**Figure 3:**
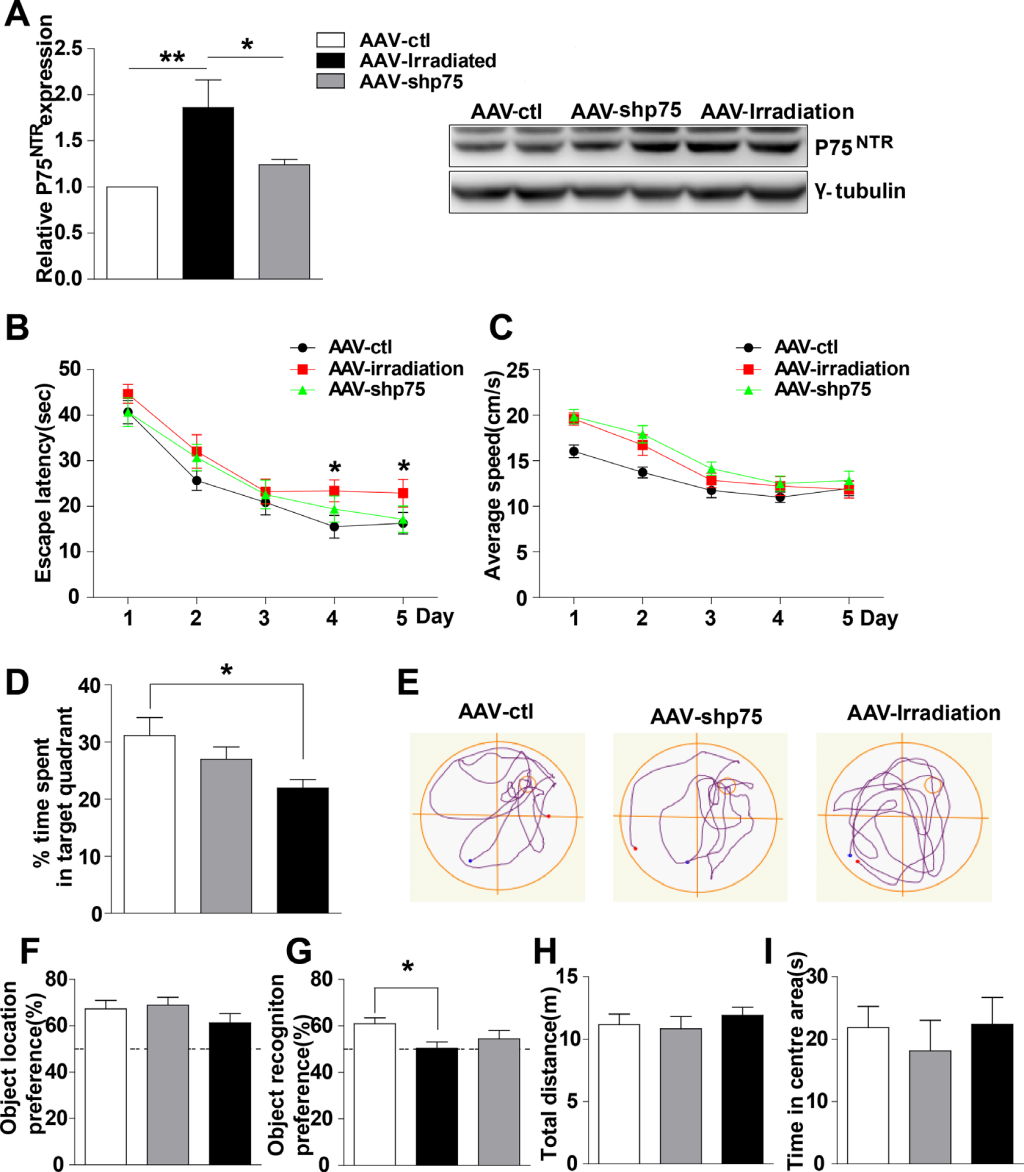
Knockdown of p75^NTR^ in hippocampus rescues spatial and nonspatial memory deficits in irradiated rats **(A)** The levels of p75^NTR^ in hippocampus extracts from AAV-ctl, AAV-irradiation and AAV-shp75 rats after virus-injection 1 month were detected. Right: Representative immunoblots. **(B-E)** *Morris Water Maze test*. Escape latencies (B), average swimming speeds (C) the percentage of target quadrant exploring time (D), and representative images of swimming paths (E) are shown. **(F)** *Object location recognition test*. No significant differences were detected in the times spent exploring a novel location among groups. **(G)** *Novel object recognition test*. AAV-irradiation rats showed worse retention performance than AAV-ctl rats. **(H-I)** *Open field test*. No significant differences were detected in the mean distance (H), and the percent time in the centre (I) among groups. All histograms represent mean ± SEM. *p<0.05; **p<0.01. n=15-20/group.

Since hippocampal p75^NTR^ overexpression imitated memory deficits, we wondered whether specifically reducing the hippocampal p75^NTR^ overexpression induced by irradiation was sufficient to prevent such memory deficits. To address this question, adenovirus expressing scramble shRNA p75 (AAV-irradiation), shRNAp75 (AAV-shp75) was infused bilaterally in the rats’ dorsal hippocampus after they were irradiated for 2 months, and 1 month later, memory function was evaluated (Figure 3). First, we assessed the efficacy of shRNAp75 in knocking down p75^NTR^. Western blot analysis showed a significant decrease of 33.3% in p75^NTR^ levels in AAV-shp75 rats compared with AAV-irradiation rats (Figure 3A). Next, memory function was assessed. The AAV-shp75 group showed a complete reversal of its spatial and recognition memory deficits. In the Morris water maze test, AAV-irradiation rats exhibited longer latency than those in the AAV-ctl and AAV-shp75 groups on day 4 of place navigation performance (Figure 3B). The changes in escape latency were not due to differences in swimming speed, which were no difference across groups (Figure 3C). In the spatial probe trial, AAV-irradiation rats spent less time in the target quadrant and exhibited shorter swimming paths to locate the target, when compared to the other groups (Figure 3D and 3E). However, there was no significant difference between the AAV-ctl and AAV-shp75 groups. Given our water maze results, we questioned whether a similar pattern could be detected using the novel location and novel object recognition tests. In response to a change in location of a familiar object, all groups spent more time exploring the object in the novel location, with no significant difference between groups (Figure 3F). In the novel object recognition test, AAV-ctl and AAV-shp75 rats spent more time exploring novel objects than familiar objects, with no group difference. However, AAV-shp75 rats demonstrated less exploration of the novel object over the familiar object (Figure 3G). We also measured the anxiety levels and sensorimotor function of all rats. As expect, our data showed no group differences in exploratory activity and measures of anxiety in the open field test (Figure 3H and 3I). These results strongly suggest that increased expression of p75^NTR^ in the hippocampus of irradiated rats is the crux of radiation-induced cognitive dysfunction.

### Effects of p75^NTR^ knockdown on hippocampal neurogenesis in the irradiated rats

**Figure 4:**
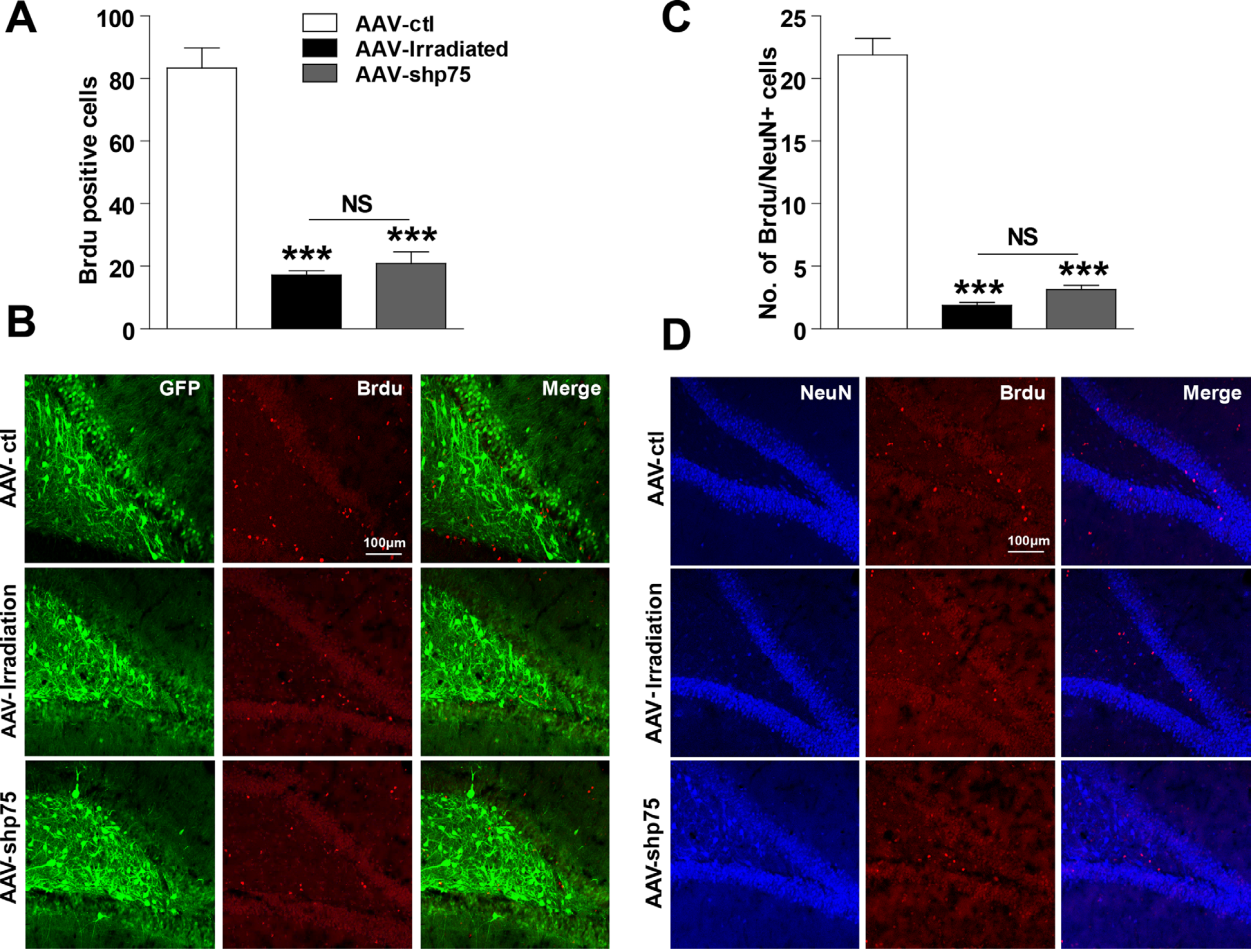
Effect of knockdown p75^NTR^ on hippocampal neurogenesis in the irradiated rats Number of BrdU-positive cells in the DG region at 3 days after BrdU treatment **(A)**, and representative confocal images of AAV-ctl, AAV-irradiation, and AAV-shp75 rats immunostained for BrdU **(B)**. Number of NeuN- and BrdU-positive colabelled cells in the DG region at 21 days after BrdU treatment **(C)**, and representative confocal image of AAV-ctl, AAV-irradiation, and AAV-shp75 rats immunostained with NeuN and BrdU **(D)**. All histograms represent mean ± SEM. ***p<0.001; NS: not significant. n=3-5/group.

Above, we demonstrated that knockdown of p75^NTR^ expression in the irradiated rat hippocampus prevents learning and memory impairments. Because altered neurogenesis and aberrant synaptic plasticity have been proposed as underlying mechanisms of radiation-induced cognitive dysfunction, we next examined whether this improvement was accompanied by hippocampal neurogenesis recovery. BrdU labeling revealed that compared to AAV-ctl rats, AAV-irradiation and AAV-shp75 rats had approximately 79.4% and 75.0% reductions, respectively, in cell proliferation in the DG at 3 months post-irradiation, and there was no significant difference between the AAV-irradiation and AAV-shp75 groups (Figure 4A and 4B). Furthermore, we used BrdU colabeling with NeuN to determine the survival and fate of the new cells. Confocal images were used to quantify the percentage of BrdU^+^/NeuN^+^ cells. BrdU^+^/NeuN^+^ cells were almost absent at 3 months following cranial irradiation, their numbers were reduced by 91.3%. This downward trend still showed no obvious improvement even though the increased expression of p75^NTR^ had been knocked down (Figure 4C and 4D).

### Effects of p75^NTR^ knockdown in the irradiated rats on dendritic spines and synapse-related proteins

**Figure 5:**
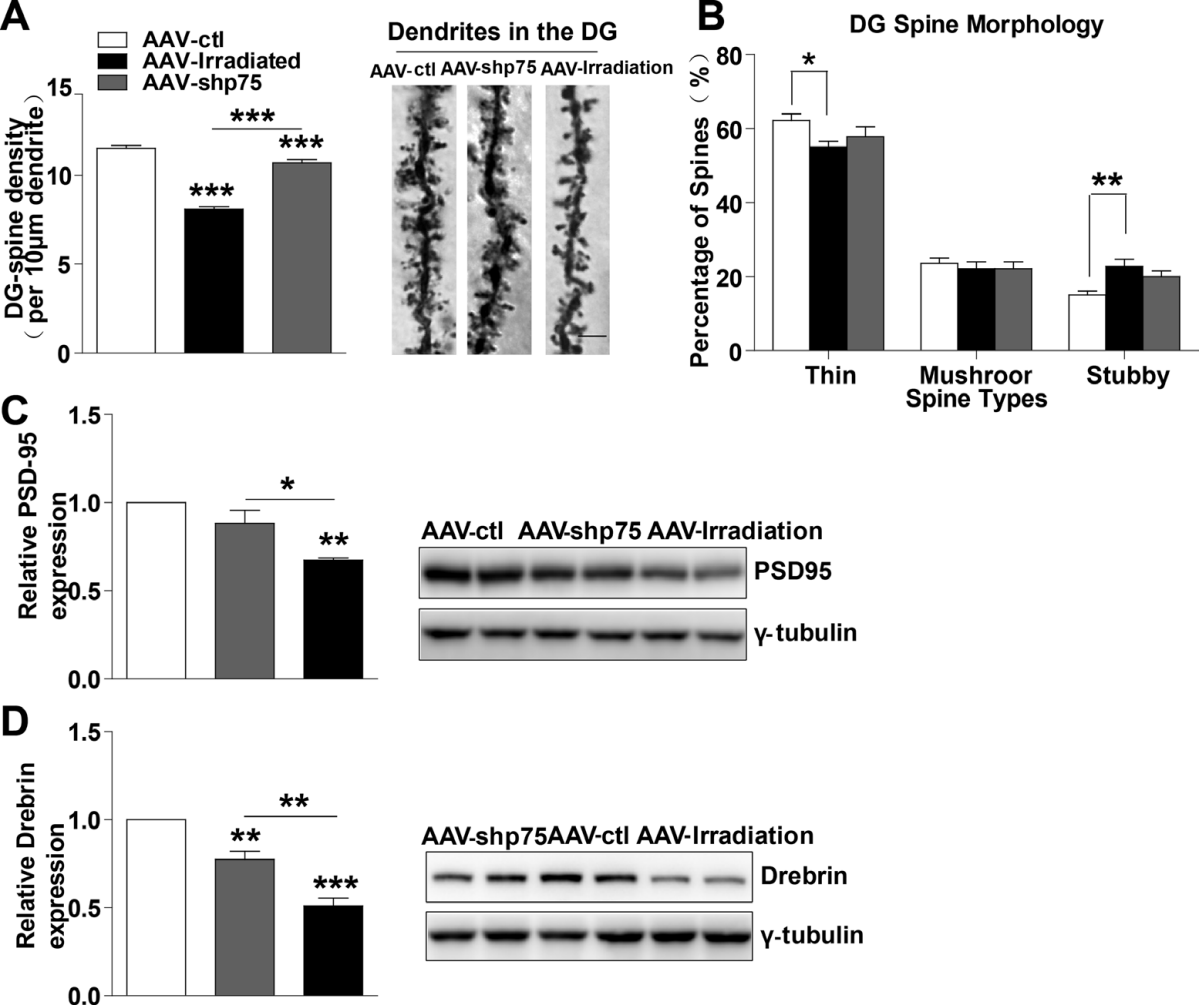
Normalization of p75^NTR^ levels in irradiated rats prevents dendritic spines and synapse-related proteins deficits **(A)** Quantitative analysis showing dendritic spine density in DG region. Right: Representative dendrites of DG granule neurons from AAV-ctl, AAV-irradiation, and AAV-shp75 rats after virus-injection 1 month. **(B)** Quantified spine types of dendritic spine including thin, mushroom, and stubby morphologies in DG from AAV-ctl, AAV-irradiation, and AAV-shp75 rats after virus-injection 1 month. Western blot for PSD-95 **(C)** and Drebrin **(D)** in total hippocampus extracts from AAV-ctl, AAV-irradiation, and AAV-shp75 rats after virus-injection 1 month. All histograms represent mean ± SEM.*p<0.05; **p<0.01; ***p<0.001. n=3-5/group.

Dendritic spines have been proposed to mediate synaptic plasticity, and changes in their density and structure clearly track with synaptic plasticity, which in turn positively correlates with cognitive function [23]. Because normalization of p75^NTR^ levels after irradiation prevented memory impairments, we wondered whether this amelioration was accompanied by spines alteration. In this context, we investigated changes in spine density and morphology, quantified via Golgi staining in DG granule neurons after a single dose of 10 Gy. AAV-irradiation and AAV-shp75 displayed a significant decrease in spine density compared with AAV-ctl (irradiation: 29.8%, shp75: 10.2%). Normalization of p75^NTR^ levels partially prevented the decay in spine density in the DG region after irradiation, and there was a significant difference between the AAV-irradiation and AAV-shp75 groups (Figure 5A). To elucidate whether increased p75^NTR^ expression could alter dendritic spine morphology, dendritic spine type was assessed. AAV-irradiation rats exhibited altered spine distribution with a significant decrease in the proportion of thin spines (11.5%), and a remarkable increase in stubby spines (52.4%). Surprisingly, AAV-shp75 rats presented the same trend as the AAV-irradiation rats in the proportions of spine types, but no significant differences were observed between AAV-shp75 and AAV-ctl groups (Figure 5B). Altogether, these results showed that cranial irradiation not only reduced dendritic spine density but also led to a shift in the spine morphology. Importantly, these dendritic changes were reversed by the reduction of aberrant p75^NTR^ levels.

Decreased levels of synapse-related proteins have been associated with memory impairments and aberrant synaptic plasticity [24, 25]. Thus, we next detected several synapse-related proteins in the hippocampus. Interestingly, the levels of synapse-related proteins such as PSD-95 and Drebrin were significantly lower in AAV-irradiation rats, a reduction that was prevented by normalization of p75^NTR^ levels (Figure 5C and 5D). These findings suggest that memory deficits in irradiated rats involve dysregulation of synapse-related proteins and that normalization of p75^NTR^ levels prevents such alterations.

### Possible mechanism

**Figure 6:**
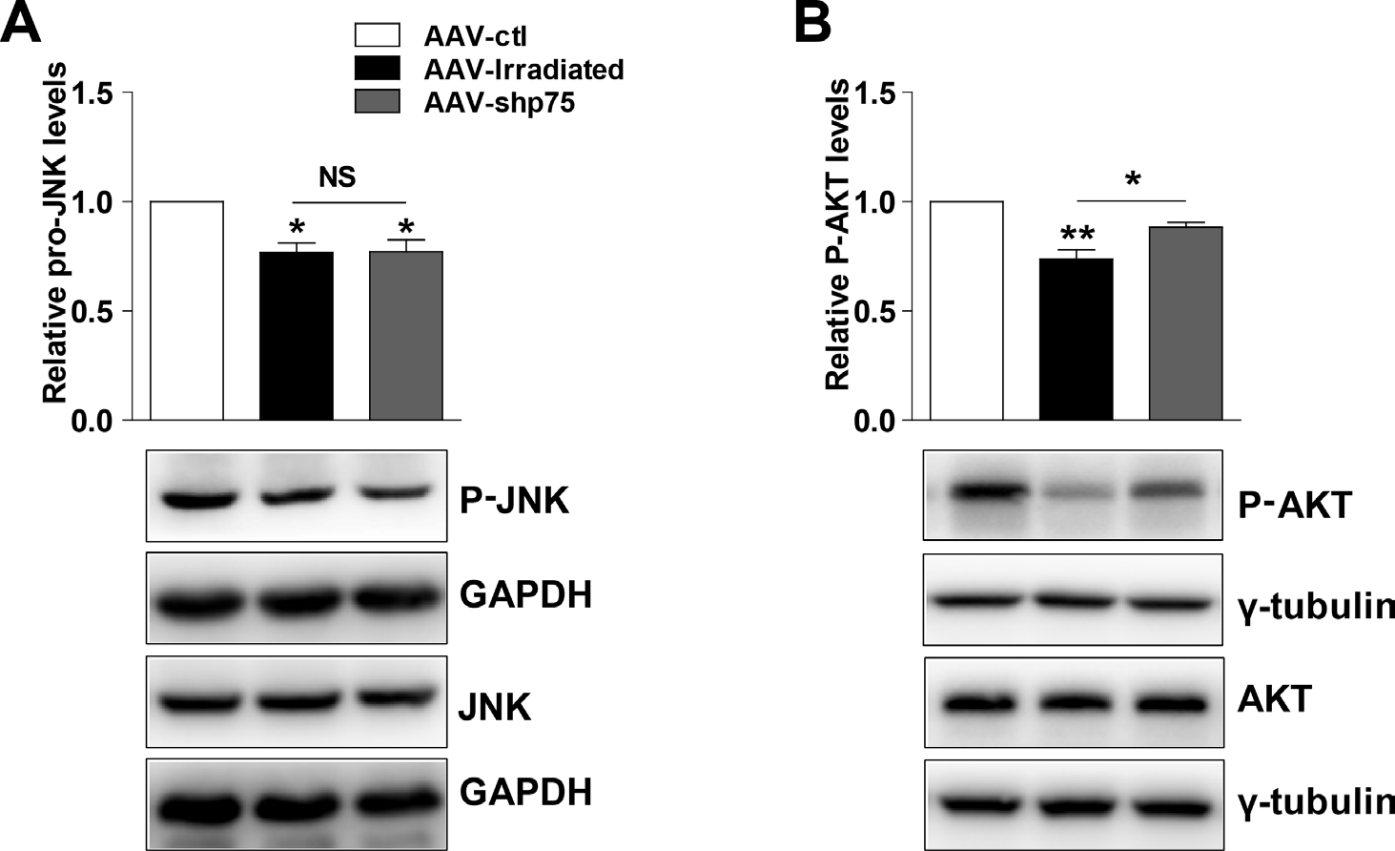
Representative western blots showing total and phosphor-JNK **(A)**, total and phosphor-AKT **(B)** in total hippocampus extracts from AAV-ctl, AAV-irradiation, and AAV-shp75 rats. All histograms represent mean ± SEM. *p<0.05; **p<0.01; NS: not significant. n=3-5/group.

The Akt pathway plays a role in the regulation of cognitive processes, and Akt activity is modulated by p75^NTR^ signaling. Thereby, we hypothesized that aberrant p75^NTR^ expression could contribute to cognitive dysfunction by altering Akt activity. We detected the Akt and phosphor-Akt protein levels after irradiation exposure. Consistent with our hypothesis, AAV-irradiation rat hippocampus showed a significant decrease in phosphor-Akt protein levels compared with AAV-ctl, and were restored in the AAV-shp75 rats (Figure 6A). Furthermore, we also tested for changes in the c-Jun N-terminal kinase (JNK) pathway. Although a reduction in phosphor-JNK protein levels was observed in AAV-irradiation rat hippocampus, no amelioration was detected between AAV-irradiation and AAV-shp75 groups (Figure 6B). These experiments suggest that pathological increase of p75^NTR^ in the irradiated rat hippocampus results in aberrant Akt activity and consequently cognitive dysfunction.

## DISCUSSION

In the current study, we demonstrated that p75^NTR^ mediates radiation-induced learning and memory dysfunctions, and corroborated previous studies implicating p75^NTR^ as a negative modulator of hippocampal function [26, 27]. Our data revealed a negative correlation between p75^NTR^ expression in the hippocampus of irradiated rats, and synaptic function such as reduced dendritic spine density, abnormal spine morphology, decreased synapse-related proteins, and memory impairments, whereas it was not involved in the process of hippocampal neurogenesis. In addition, overexpression of p75^NTR^ in the hippocampus of normal rats mimicked radiation-induced learning and memory deficits, while specific knockdown the p75^NTR^ levels in the hippocampus of irradiated rats prevented such cognitive impairments.

P75^NTR^ has been shown to be important for the initiation of apoptotic cell death in developing neurons [28]. Recent studies have shown that p75^NTR^ plays an important role in hippocampal neurogenesis and synaptic plasticity [29, 30]. In the present study, we found that p75^NTR^ is upregulated in the hippocampus after cranial irradiation, accompanied by hippocampal-dependent spatial and nonspatial memory impairments. Given these data, we wondered whether normalization of p75^NTR^ expression in the irradiated rat hippocampus would prevent memory declines. In agreement with our hypothesis and exploring a role of aberrant hippocampal p75^NTR^ levels, adenoviruses expressing shRNAp75 and overexpression p75^NTR^ were proposed to infuse bilaterally in the dorsal hippocampus. Thus, the localization of p75^NTR^ in the hippocampal cells should be determined. Previous laboratory studies have demonstrated p75^NTR^ is expressed by astrocytes in addition to neurons, especially during development and after injury [31, 32]. However, Brito V et al. recently reported that neuronal p75^NTR^ upregulation underlies hippocampal dysfunction in Huntington's disease [22]. Our results showed that p75^NTR^ immunoreactivity co-expresses with NeuN before and after irradiation, with no overlap between GFAP and p75^NTR^ in the hippocampus, suggesting that p75^NTR^ is predominantly located in hippocampal neurons. These observations are consistent with the results of the Brito V et al. study. Moreover, specific upregulation of p75^NTR^ in hippocampal neurons reproduced memory impairments, further supporting the conclusion that p75^NTR^ in the hippocampal neurons of irradiated rats is responsible, at least partially, for the memory impairments.

We found that knockdown of p75^NTR^ expression in the irradiated rat hippocampus prevented spatial and nonspatial memory declines, ameliorated spine abnormalities, and normalized synapse-related proteins, further supporting a crucial role for p75^NTR^ in radiation-induced cognitive deficits. From these data, it is unclear whether only the increase of p75^NTR^ in the hippocampus is responsible for radiation-induced dysfunctions, or whether upregulation of p75^NTR^ levels in other brain areas also contributes to this process. To address this question, we evaluated p75^NTR^ expression in the PFC, which is associated with cognition. Interestingly, there was no change in the PFC before and after irradiation. This finding may further supports the role of p75^NTR^ in the hippocampus in radiation-induced cognitive deficits. It is worth noting that selectively increasing p75^NTR^ levels only in the normal rat hippocampus could reproduce memory deficits. To the best of our knowledge, these findings are the first to show that p75^NTR^ in the hippocampus negatively modulates learning and memory abilities after irradiation.

Our experiments showed that increased p75^NTR^ protein levels were accompanied by increased p75^NTR^ transcripts (Supplementary Figure 2A), but the mechanism is poorly understood. Recently, it has been reported that TAp73 is a direct transcriptional activator of p75^NTR^ [33], which prompted us to investigate whether increased p75^NTR^ expression was related to higher hippocampal TAp73 levels. TAp73 is a transcription factor belonging to the p53 family, whose members share similarities in sequence and function [34, 35]. Moreover, DNA damage caused by ionizing radiation can promote p73 transcription activity [36]. In accordance with these findings, we observed a significant increase in TAp73 protein levels in parallel post-irradiation (Supplementary Figure 2B), supporting the view that TAp73 could induce the increase in p75^NTR^ mRNA after irradiation, and suggesting that the TAp73/p75^NTR^ axis may underlie the pathology of radiation-induced cognitive dysfunction. However, we cannot exclude the possibility that other transcription factors might also be involved in the process of regulating p75^NTR^ expression, this remains to be studied. We further aimed to elucidate the molecular mechanism of aberrant hippocampal p75^NTR^ expression.

Neurogenesis plays an important role in the pathogenesis of cognitive dysfunction after irradiation. To evaluate whether p75^NTR^ was involved in this process, we assessed cell proliferation by BrdU labeling. BrdU labeling revealed that compared to AAV-ctl, AAV-irradiation and AAV-shp75 rats had approximately 79.4% and 75.0% reductions, respectively, in cell proliferation at 3 months post-irradiation. This indicated that very little, if any, recovery was seen with respect to cell proliferation in DG after treatment. Furthermore, we used BrdU plus NeuN to determine the survival and fate of the new neurons. The percentage reductions were roughly the same between AAV-irradiation and AAV-shp75, regardless of treatment. We found that overexpression of p75^NTR^ was not involved in the process of neurogenesis impairments induced by irradiation, at least in this model. One possible explanation is that the overexpression of p75^NTR^ cannot activate the JNK pathway. Accumulating evidence has emerged demonstrating that activation of JNK signaling is essential for mediating neuronal apoptosis and cell proliferation [37, 38]. Here, we found that treatment with AAV-shp75 did not increased JNK phosphorylation and had no effect on total BrdU cell number or BrdU incorporation, indicating that p75^NTR^ does not activate JNK signal transduction pathways.

In addition to hippocampal neurogenesis, synaptic plasticity also plays an important role in the pathogenesis of cognitive dysfunction after irradiation. How might the increased p75^NTR^ levels lead to dendritic spine changes? We found that in the DG, there were significant reductions in spine density and abnormal morphology distributions induced by irradiation. Additionally, we observed decreased expression of synapse-related proteins, such as PSD-95 and Drebrin. Indeed, memory improvements in AAV-shp75 rats correlated with a recovery of the changes in spines density, morphology and synaptic-proteins. Several mechanisms might help to explain how aberrant p75^NTR^ levels mediate synaptic and memory deficits in irradiated rats. First, p75^NTR^ operates as a negative regulator of dendritic spine density and morphology [39, 40]. AAV-irradiation rats show a significant loss of dendritic spines in DG neurons. Dendritic spines are the primary recipients of excitatory input in the CNS, and changes in spine density can bear responsibility for functional differences at the synaptic level [41]. Spine morphology can predict both spine stability and synaptic strength, and structural plasticity of spines is related to learning and memory abilities [42]. Thin spines maintain structural flexibility and can accommodate new, enhanced or recently information, making them candidate ‘learning spines’ [43]. By decreasing the proportion of learning spines, radiation may decrease a neuron's ability to form new synapses. In contrast with the decrease in the fraction of thin spines, a marked increase in the proportion of stubby spines was observed in DG after radiation exposure. It has been reported that dopamine receptors are located on the spine neck [44], and dopaminergic processes are relevant to cognitive function, while stubby spines lack a neck. It can be speculated that a significant increase in the proportion of stubby spines after radiation exposure might lead to alterations in dopaminergic signaling which in turn induce cognitive changes. Aberrant spine changes could be recovered by knockdown of p75^NTR^ levels. We confirmed that aberrant p75^NTR^ expression could contribute to reduction in the number and complexity of hippocampal dendritic spines. Such changes may have long-term consequences for radiation-induced cognitive deficits. Second, our data showed that p75^NTR^ regulates various synapse-related proteins. PSD-95 plays a major role in regulating synaptic plasticity, and it has been shown to be associated with synapse number or synaptic loss. Drebrin is a f-actin postsynaptic binding protein that is associated with synaptic plasticity [45]. Our experiments showed that cranial irradiation decreased the levels of cytoskeletal proteins PSD-95 and Drebrin. Decreased levels of synapse-related proteins may be associated with memory impairments and aberrant synaptic plasticity. In accordance, memory improvements correlated with a recovery of the expression of synapse-relative proteins in AAV-shp75 rats. Thus, we can be reasonably sure that p75^NTR^ represents a potential regulator of radiation-induced synaptic pathology. These findings suggest that adequate p75^NTR^ levels are required for normal forms of synaptic plasticity and cognitive processes, while dysregulation of p75^NTR^ will activate certain transduction pathways for memory processes.

How might increased p75^NTR^ levels result in cognitive dysfunction? It has been reported that p75^NTR^ could modulate a number of intracellular pathways including those of Akt, NF-κB, MAPKs, JNK, RhoA, PKA and HIF [46]. Akt is important for the regulation of a diverse array of biological effects, including cell proliferation, survival and metabolism. Recently, studies have shown that dysregulation of Akt leads to synaptic plasticity alternation and modulation of the autophagic process in neuronal protection, which are related to cognitive function [47–49]. Interestingly, we found decreased Akt activity in the hippocampus of AAV-irradiation rats compared with AAV-ctl and AAV-shp75 groups, suggesting that a pathological increase of p75^NTR^ in the irradiated rat hippocampus results in aberrant Akt activity and, consequently, cognitive dysfunction. In this study, we are only present a preliminary exploration of the effects of Akt signaling pathways, and the precise mechanism needs further research. We cannot rule out that some other signaling molecules such as the small GTPase RhoA and NF-κB that are known to be regulated by p75^NTR^ might also contribute to synaptic plasticity and cognitive dysfunction. Further work remains to be done in this field.

In conclusion, the present findings demonstrate that p75^NTR^ upregulation in the hippocampus contributes to radiation-induced cognitive deficits. This work provides validation for development of individualized therapies involving p75^NTR^ to treat synaptic and memory impairments, and may help to reduce the number of people affected by radiation-induced cognitive dysfunction in the coming years.

## MATERIALS AND METHODS

### Animals

Twenty-one-day-old male SD rats (50-60 g) were obtained from the Medical Experimental Animal Center of Soochow University (Suzhou, China). All experiments were performed in accordance with federal and institutional guidelines and approved by the Animal Care and Ethics Committee of Soochow University, China. In the behavioral test, the rats were randomly divided into two parts: one part is only for the Morris water maze test, and the other part is for the open filed test, novel object and location recognition test in order.

### Irradiation

The rats were anesthetized with 3.6% chloral hydrate (360 mg/kg) and placed in a prone position as previously described [12, 50]. Prior studies have shown that cognitive impairments can be induced by a single dose of 10 Gy [51]. The whole brain of each rat received a single dose of 0 (control group) or 10 Gy (irradiation group) of a 4-MeV-electron beam. The 4-MeV-electron beam was generated by a linear accelerator (SL 18, Philips, UK) with a cone size of 25 cm × 25 cm and a dose rate of 210-220 cGy/minute.

### Morris water maze test

The Morris water maze test was performed as described previously [50]. All rats performed 4 trials per day. The platform remained at a constant location for all trials, but the animal's start location changed on each trial. Each rat was allowed 60 s to locate the platform. The rat was permitted to rest on it for 10 s before being assisted back into its home cage. If the rat failed to find the platform in 60 s, it was guided to the platform and allowed to remain on the platform for 10 s. A spatial probe test, in which the platform was removed and rats were allowed to swim freely for 60 s to find the platform, occurred on day 6. The time to reach the platform, path length, swim speed and number of crossing the target zone were recorded.

### Open field test

The open field box consisted of a square black box (length 45 cm× width 45 cm× height 60 cm). Each animal was placed in the box for 10 min. After 1 h, the rat was allowed to explore the arena for 5 min and the time spent in the center of the arena (length 22 cm × width 22 cm) was recorded.

### Novel object recognition

In the training trial, the subject was presented with a pair of identical objects that had been placed in the opposite corners for 10 min. Exploration of the objects was considered to occur when the rat showed any exploratory behavior (orienting its head toward the object, sniffing the object, or entering an area within 1 cm around the object). In the testing trial (performed 1 h later), one of the familiar objects was exchanged with a novel object. The rat was left in the cage for 5 min. The exploration times spent on the familiar and the novel object during the test phase were recorded.

### Object location recognition

The rat was habituated to an open-top cage (length 45 cm× width 45 cm× height 60 cm) in which two identical objects had been placed in the opposite corner for 10 min, and the time interacting with the objects was measured. One hour later, one of the objects was moved while the other one was left in the same spatial position. Time exploring each object was recorded.

### Western blot analysis

Tissue homogenates were lysed in RIPA buffer (150 mM NaCl, 1% NP-40, 0.5% DOC, 0.1% SDS, 50 mM Tris, pH, 8.0) and subjected to 10% SDS–PAGE. Protein concentrations were determined using the BCA Kit (Thermo). Antibodies used were anti-p75^NTR^ (1:5000; Abcam); anti-TAp73 (1:200, Santa Cruz Biotechnology); anti-PSD95 (1:2000; Abcam); anti-Drebrin (1:1000; Abcam); anti-JNK and anti-phospho-JNK (1:1000; Ruiying Biological); anti-Akt and anti-phospho-Akt (1:1000; Ruiying Biological); anti-γ-tubulin (1:10000; Sigma-Aldrich); GAPDH (1:5000; Beyotime).

### Adeno-associated virus and stereotaxic injection

For knockdown p75^NTR^, shRNA oligomers targeting mouse p75^NTR^ were purchased from Genechem (Shanghai, China). Adeno-associated virus (serotype 8, AAV8), which encodes GFP as well, expressing shRNA targeting mouse p75 (AAV-shp75) or mouse p75^NTR^ (AAV-p75) were generated by the Genechem Co., Ltd. (Shanghai, China). AAV8 expressing scrambled shRNA p75 (AAV-ctl) or GFP alone served as controls. Both AAV-shp75 and AAV-irradiation are conditions in which rats have been irradiated but in the first one p75^NTR^ has been knockdown while in the second one scramble has been used. Following anesthesia with 3.6% chloral hydrate (360 mg/kg), rats were infused bilaterally in the dorsal hippocampus (-3.7 mm anteroposterior from bregma, ±2.2 mm mediolateral from bregma and 3.5 mm below the surface of the skull). AAVs were injected at the rate of 0.2 μl/min, leaving the cannula in place for 5 min to ensure complete diffusion of the virus, after which it was slowly retracted from the brain (n=15-20 per group). The animals were then returned to the housing facility for 30 days after waking up.

### Golgi staining

For spine analyses, Golgi staining was performed using the FD Rapid GolgiStain™ Kit (FD Neurotechnologies Inc.), following the manufacturer's guidelines. The neurons that satisfied the following criteria were chosen for analysis in each of the experimental groups: (1) presence of untruncated dendrites; (2) consistent and dark Golgi staining along the entire extent of the dendrites; and (3) relative isolation from neighboring neurons to avoid interference with analysis [52]. Three to five dendritic segments, each at least 30 μm in length on secondary or tertiary dendritic segments, were analyzed per neuron, and a total of 10–11 neurons were analyzed per brain. On the basis of morphology, spines were classified into the following categories: (1) Thin: spines with a long neck and a visible small head; (2) Mushroom: big spines with a well-defined neck and a very voluminous head; and (3) Stubby: very short spines without a distinguishable neck and stubby appearance [52]. Image J software was used to calculate linear spine density, which was presented as the number of spines per 10 μm of dendrite length.

### BrdU labelling and tissue processing

Following irradiation, rats received an i.p. injection (100 mg/kg/day) of 5-bromo-2’-deoxyuridine (BrdU, Sigma) twice a day for 7 consecutive days. Three weeks after the conclusion of the BrdU injections, rats were anesthetized and perfused with ice-cold saline followed by ice-cold 4% paraformaldehyde. Brains were removed and post-fixed overnight in 4% paraformaldehyde and then equilibrated in 30% sucrose. Free-floating 30-μm-thick sections of the brains were then cut through the entire hippocampus on a freezing microtome.

### Immunofluorescence

For detection of BrdU-labeled nuclei, sections were incubated with 1N HCl at 45°C for 30 min, and then neutralized in 0.1 M borate buffer (PH 8.5) for 10 min. Sections were washed 3 times with PBS and then blocked using 10% calf serum for 2 h at room temperature. After blocking, sections were incubated overnight with mouse monoclonal anti-BrdU antibody (1:300; Biolegend) at 4°C. Co-labeling runs used rabbit anti-NeuN (1:500; Abcam) for the visualization of neurons. For immunofluoresence staining with Brdu, sections were incubated with primary antibodies as follows: rabbit anti-p75 (1:100; Ruiying Biological), mouse anti-NeuN (1:100; Santa Cruz Biotechnology) and mouse anti-GFAP (1:100; Santa Cruz Biotechnology). Sections were washed 3 times in PBS and incubated with appropriate fluorescence-conjugated secondary antibodies as follows: Alexa Fluor 555 donkey anti-mouse, Alexa Fluor 488 donkey anti-rabbit, and Alexa Fluor 405 Goat anti-rabbit IgG (1:500; Biolegend). Sections were mounted after washing with PBS and visualized with confocal microscopy.

### Statistical analysis

Data were collected from at least 3 independent experiments. T-tests and One-Way *ANOVA* for independent samples were performed using SPSS 16.0 software. Where behavioral data were non-normally distributed, we used a Mann-Whitney U-test. Values are presented as the mean ± SEM. Significance in differences was accepted at *P*<0.05 (*P<0.05, **P < 0.01, and ***P < 0.001).

## SUPPLEMENTARY MATERIALS FIGURES


