# Supplementary material for: The p75 neurotrophin receptor regulates cranial irradiation-induced hippocampus-dependent cognitive dysfunction
Source: Oncotarget. 2017 Mar 23;8(25):40544–57. doi: 10.18632/oncotarget.16492 (PMC5522261; doi:10.18632/oncotarget.16492)
Supplement: Supplementary file 1 [file oncotarget-08-40544-s001.pdf]

The p75 neurotrophin receptor regulates cranial irradiation-induced hippocampus-dependent cognitive dysfunction

SUPPLEMENTARY MATERIALS

MATERIALS AND METHODS

Reverse transcription polymerase chain reaction (RT-PCR)

RNA was extracted from tissues using Trizol Reagent (Invitrogen). RT reactions were performed with the RevertAid First Strand cDNA Synthesis Kit (Promega). The following primers were used. The primers for P75NTR: GAGGGCACATACTCAGACGA (forward), CTCTTCGCATTCAGCATCAG(reverse); the primers for GAPDH: CAAGGTCATCCATGACAACTTTG (forward), GTCCACCACCCTGTTGCTGTAG (reverse).

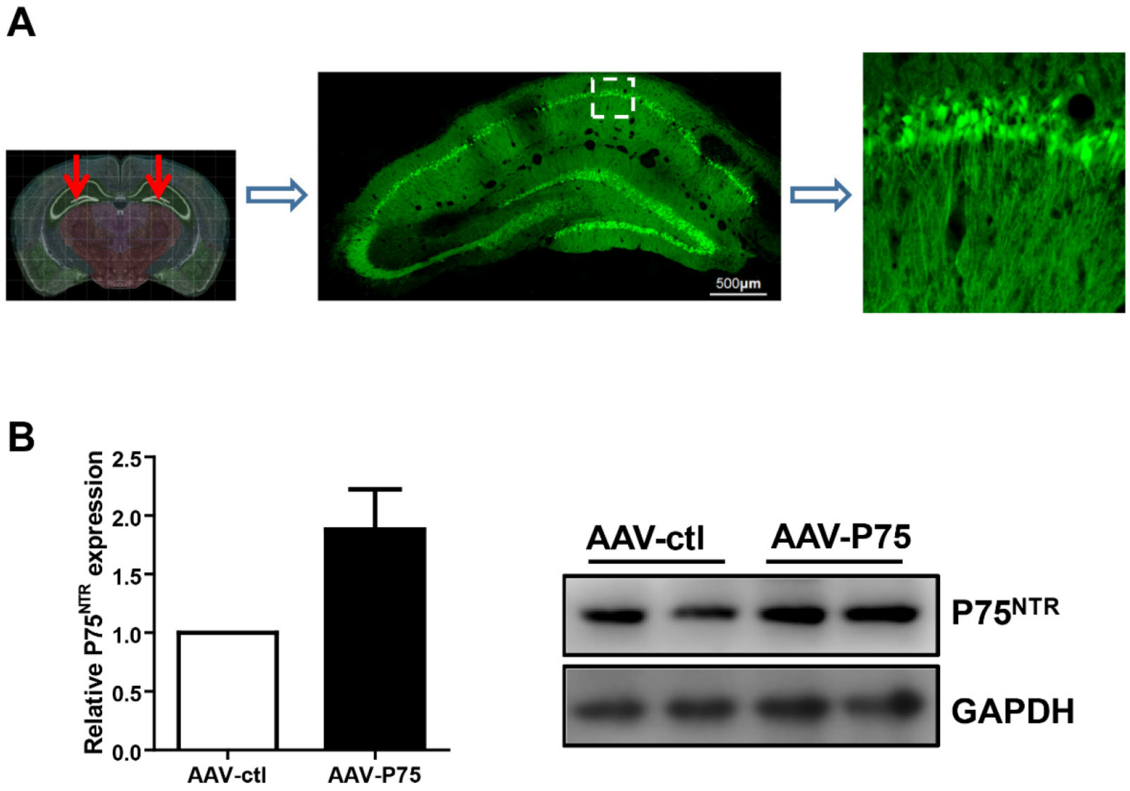

Supplementary Figure 1: (A) Representative confocal microscopy image (low magnification) and Magnified images (right) showing the rat hippocampus injected with AAV8 encoding GFP. **(B)** Western blot analysis of $p75^{NTR}$ and γ-tubulin as loading control in total hippocampus extracts from AAV-ctl and AAV-p75 rats after virus-injection 1 month, showing increased $p75^{NTR}$ levels in AAV-p75 rats compared with AAV-ctl rats. Right: Representative immunoblots.

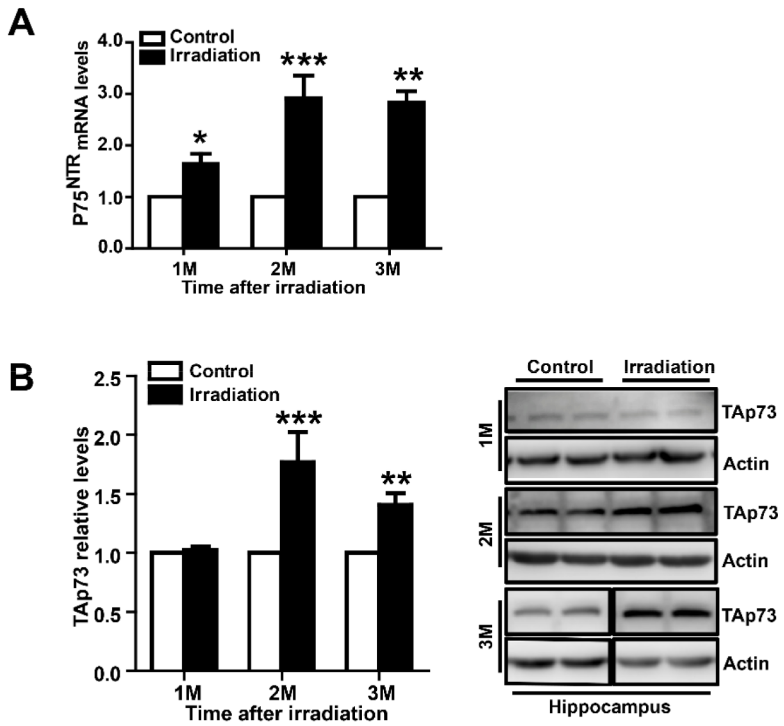

Supplementary Figure 2: Increased hippocampal p75^{NTR} mRNA expression associates with higher levels of TAp73 in the irradiated rats hippocampus. (A) Histograms showing p75^{NTR} mRNA expression analyzed by RT-PCR in the hippocampus after irradiation. **(B)** Western blot for TAp73 in hippocampus at 1, 2 and 3 months from control and irradiated SD rats. Actin was used as loading control. Right: Representative immunoblots. Data are presented as mean +SEM. $^{**}p<0.01$; $^{***}p<0.001$. n=5/group. Independent samples t-test.
